# Editorial: Brain Modeling of Neurogenerative Disorders

**DOI:** 10.3389/fninf.2022.937790

**Published:** 2022-05-26

**Authors:** Jorge Gulín-González, Maria L. Bringas-Vega, Eduardo Martínez-Montes, Petra Ritter, Ana Solodkin, Mitchell Joseph Valdes-Sosa, Pedro Antonio Valdes-Sosa

**Affiliations:** ^1^Facultad CITEC, Centro de Estudios de Matemática Computacional (CEMC), Universidad de las Ciencias Informáticas (UCI), Havana, Cuba; ^2^The Clinical Hospital of Chengdu Brain Sciences Institute, MoE Key Lab for Neuroinformation, University of Electronic Sciences and Technology of China, Chengdu, China; ^3^Cuban Neuroscience Center, Havana, Cuba; ^4^Section Brain Stimulation, Charité - Universitätsmedizin Berlin, Berlin, Germany; ^5^School of Behavioral and Brain Sciences, University of Texas, Dallas, TX, United States

**Keywords:** brain modeling, neurogenerative disorders, system biology, Machine Learning, Deep Learning, multiscale modeling

Modeling is now recognized as an essential tool for studying one of the most complex structures known: the human brain. Since the seminal work of Wilson and Cowan in 1970s, who introduced the first neural mass model, much effort has been dedicated to understanding the brain dynamics and elucidating their intricate relation to neurodegenerative diseases or other aging-related brain disorders. Advances in Neurosciences have been driven by the vertiginous growth in all aspects of multi-omic data: genetic, epigenetic, transcriptomics, connectomics, exposomics, and phenomics. The continuous growth in computational resources has matched such overwhelming amounts of data. Since the phenomena/processes linked to diseases and/or brain disorders occur at different time-space scales, complex multi-scale models were needed and developed. A cornerstone of Brain modeling is high-dimensional computational simulation. General and specific-purpose software platforms for neural and brain network construction and simulation have been developed for this purpose. Examples of these are The Virtual Brain platform (TVB) (www.thevirtualbrain.org), Blue Brain (https://portal.bluebrain.epfl.ch), and The Budapest Reference Connectome (https://pitgroup.org/connectome/), The Neural Simulation Technology (NEST) (https://www.nest-initiative.org/) and The General Neural Simulation System (GENESIS) (http://genesis-sim.org/). A current tendency in neuroscience is to combine personalized brain network modeling adapted to specific persons with Artificial Intelligence techniques, e.g., Machine Learning (ML) and Deep Learning (DL), to build predictive models of brain function and dysfunction.

This Research Topic is inspired by the BrainModes conference series (https://brainmodes.org/) that is dedicated to the above-outlines topic. It provides a collection of six publications highlighting the latest trends in this challenging and dynamic field. It includes two Reviews, three Original Research, and one Brief Research Report. These cover significant scopes of brain modeling focused on explaining relevant characteristics of brain diseases or aging-related brain disorders.

The two Reviews summarize knowledge about novel topics of computational neurosciences relevant to the study of neurodegeneration and aging. Stefanovski et al. reviewed the use of computational models for multi-scale brain simulations to improve Alzheimer's Disease (AD) diagnostics, mainly in the early stages of dementia. The authors outline the existing computational models for AD that aim to provide a mechanistic understanding of the disease and they conceptualize how multi-scale modeling can link molecular aspects of neurodegeneration in AD with large-scale brain network modeling using TVB platform. In the other review, González et al. explore some of the most relevant models of astrocyte metabolism, including genome-scale reconstructions and astrocyte-neuron interactions developed in previous research (Schuster et al., 2000; Agutter, 2005). The subject of this review contrasts with preceding ones focused on computational models regarding calcium signaling in astrocytes and its modulation in synaptic transmission, gliotransmitter release, and related processes (Oschmann et al., 2016; Manninen et al., 2019). Additionally, and not less importantly, the authors discuss strategies from the multi-omics viewpoint and computational models of other glial cell types, thus increasing our understanding of brain metabolism and its association with neurodegenerative diseases.

In the Original Research by Castellazzi et al., ML is leveraged to elucidate a critical aspect of neurodegenerative diseases, namely, the correct disease diagnosis. The study concerns two frequent dementias, AD and vascular dementia (VD). The authors employed three kinds of ML algorithms (artificial neural network, support vector machine, and adaptive neuro-fuzzy inference system) combined with advanced magnetic resonance imaging features to distinguish VD from AD. They also developed approaches that might help predict the prevalent disease in subjects with an unclear profile of AD or VD. The approach showed a high discriminant power to classify both diseases.

Parkinson's disease (PD) is a motor disorder affecting millions of people worldwide, mostly older adults. PD is caused by loss of dopaminergic neurons in substantia nigra pars compacta (SNc), located in the midbrain region (Fu et al., 2018). However, the precise cause of cell death has not been established. For this purpose, Muddapu and Chakravarthy developed a multi-scale computational model of the subsystem of the basal ganglia comprising the subthalamic nucleus, globus pallidus externa, and SNc. They simulated the molecular pathways involved in cell death of SNc neurons in terms of detailed chemical kinetics. The authors showed that energy deficiencies occurring at cellular and network levels could precipitate the excitotoxic loss of SNc neurons in PD. Therapeutic effects of neuroprotective interventions were simulated.

Brain rhythms are critical in coordinating brain networks. Rhythms of different frequencies have specific coupling properties, such as cross-frequency coupling (CFC), which potentially provides synchronization and interaction mechanisms between local and global processes across cortical networks. Thus, CFC is a novel feature of brain activity correlated with brain function and dysfunction. Measures for CFC exist in the form of the cross-bispectrum or its normalized version, which are third-order statistical moments in the frequency domain (Chella et al., 2014). The investigation of Bartz et al. makes two contributions to analyzing brain oscillations with CFC techniques. First, a new bispectral CFC measure selective to couplings between three or more brain sources is introduced. Secondly, they present the correct empirical distribution for the coupling measure, which is necessary to assess the significance of coupling results properly. The corrected statistic is not limited to their particular measure but holds for all complex-valued coupling estimators.

Tuladhar et al. present a fascinating study focused on a paradigm for modeling neural diseases *in silico* with DL methods. The developed model is used in modeling posterior cortical atrophy (PCA), an atypical form of AD affecting the visual cortex. The authors simulated PCA in deep convolutional neural networks trained for visual object recognition by randomly injuring connections between artificial neurons. They reported that injured networks progressively lost their object recognition capability. This paradigm can be used to study the impact of neural plasticity in partially recovering the object recognition capability and can also be applied to other cognitive domains (e.g., motor control, language processing, and decision making). Thus, this research opens possibilities for DL applications in neurodegenerative brain disorders and aging.

We want to thank all authors for their cutting-edge contributions. The Research Topic provides to readers an opportunity to update their knowledge in clinically relevant computational neurosciences research.

## Author Contributions

JG-G, EM-M, PR, and PV-S wrote and edited the text. All authors contributed to the article and approved this version.

## Conflict of Interest

The authors declare that the research was conducted in the absence of any commercial or financial relationships that could be construed as a potential conflict of interest.

## Publisher's Note

All claims expressed in this article are solely those of the authors and do not necessarily represent those of their affiliated organizations, or those of the publisher, the editors and the reviewers. Any product that may be evaluated in this article, or claim that may be made by its manufacturer, is not guaranteed or endorsed by the publisher.
